# Functional Haplotype and eQTL Analyses of Genes Affecting Cadmium Content in Cultivated Rice

**DOI:** 10.1186/s12284-019-0340-8

**Published:** 2019-11-21

**Authors:** Sang-Beom Lee, Gyeong-Jin Kim, Kyu-Won Kim, Sang-Ho Chu, Jung-Du Shin, Yu-Ji Lee, Yong-Jin Park, Sang-Won Park

**Affiliations:** 1 Chemical Safety Division, National Institute of Agriculture Science (NIAS), Wanju, 55365 South Korea; 2 Department of Plant Resources, College of Industrial Sciences, Kongju National University, Yesan, 32439 South Korea; 3 Center of Crop Breeding on Omics Artificial Intelligence, Kongju National University, Yesan, 32439 South Korea; 4 Department of Climate Change and Agro-Ecology, National Institute of Agriculture Science (NIAS), Wanju, 55365 South Korea

**Keywords:** Cd-related genes, Genome-wide association study, Gene expression level, Inorganic components, Candidate gene

## Abstract

**Background:**

Rice is a major food resource for Asian countries including Korea. However, most Asian countries are facing food safety problems due to cropland contamination by heavy metals. Thus, this study was conducted to investigate genetic factors affecting the expression of cadmium (Cd) gene, and to confirm differences in Cd translocation among cultivars because the current molecular understanding of Cd uptake-transport mechanisms remains insufficient. Associations between genotypes and gene expression level of Cd-related genes such as NRAMP, MTP, and HMA gene families in the rice core collection were analyzed at the genomic level.

**Results:**

Os01g0956700, Os05g0128400 and Os11g0485200 showed strong associations between expression level and genotype in the rice core collection, the regulatory factors that associated with these genes in *cis* and *trans* were founded. The association between the expression level and genotype of the candidate gene (Os01g0611300: metal tolerance protein) predicted to affect Cd content in rice by a previous genome-wide association study (GWAS) was also analyzed. Furthermore, as a result of the phylogeny and haplotype analyses of the candidate gene, high-Cd tolerance cultivars were selected. The correlations between Cd and other inorganic components (Mg, Mn, Fe, Cu and Zn) in the roots, stems, leaves and unpolished grain of selected rice cultivars were analyzed.

**Conclusion:**

Therefore, these results may be useful for understanding the uptake-transport mechanisms of Cd and other inorganic components via molecular genetics and may help rice breeders develop new low-Cd cultivars in the near future.

## Background

Cadmium (Cd) is a toxic material in the environment that threatens living organisms including humans and staple crops, through natural circulation in the food chain (Ogawa et al. 2009). In particular, Cd toxicity is a potential result of chronic low exposure level (Clemens et al. 2013). Cd is released into the environment from phosphate fertilizers, polluted irrigation water, waste incinerators and abandoned mine tailings, and Cd pollutes most croplands (Di Toppi and Gabbrielli 1999; McGrath et al. 2001). To date, studies from around the world have reported on Cd uptake by plants in the fields of molecular biology, plant physiology and breeding genetics. These studies have raised awareness about the safety of staple foods. Generally, heavy metals such as Cd are toxic, non-essential trace elements that can reduce the amounts of essential trace elements in the body and react with S and N in amino acid side chains (Clemens 2001). In addition, unlike iron (Fe) and cooper (Cu), Cd disturbs the balance of essential elements without directly catalyzing reactive oxygen species (ROS) (Stohs and Bagchi 1995; Ogawa et al. 2009). Therefore, plants have developed specific uptake, accumulation, transport, chelation and sequestration mechanisms to maintain essential elements and minimize the detrimental effects of non-essential elements (Clemens 2001; Hall and Williams 2003). Plants synthesize Cys-rich and metal-binding peptides (phytochelatins (PCs) and metallothioneins) to eliminate toxicity when exposed to heavy metals (Clemens 2001). PCs are peptides synthesized from glutathiones (GSH) by PC synthase ((γ-Glu-Cys) 2–11-Gly); Cd detoxification then occurs through a mechanism involving GSH and PCs (Mendoza-Cozatl et al. 2005). GSH is known to be involved in reductive reactions as an important defense substance against heavy metals ROS, and xenobiotics. GSH is synthesized by two adenosine triphosphate (ATP)-dependent reactions catalyzed by γ- glutamylcysteine synthetase and glutathione synthetase, which are present in the cytosol, and plant chloroplasts play a role in catalyzing these ATP-dependent reactions. The synthesis of GSH is carried out via the following steps: sulfate uptake ➔ sulfate activation (ATP sulfurylase) ➔ reduction of sulfate to sulfide (APSK, PAPSR, APSR, Sir) ➔ cysteine biosynthesis (SAT, HAT, OASIOAH TL, β-CTS and γ-CTL). The synthesized Cys provides a substrate for GSH biosynthesis (γ-ECS, GS) and phytochelatin biosynthesis (Mendoza-Cozatl et al. 2005) (Additional file 1: Figure S1). In the synthesis process, APSK utilizes various metabolites such as phytosulfokines, steroids, glucosinates and sulfated flavonols, and synthesizes PAPS (Leustek and Saito 1999).

On the other hand, mitogen-activated kinase (MAPK) is known to be activated in tandem with GSH depletion, but its biological function with respect to Cd has not yet been clarified precisely (Stohs and Bagchi 1995; Guan et al. 2016).

Depending on the redox state in the soil, the extent to which Cd is transferred to crops is variable. O_2_ in the atmosphere moves to the roots through the crop aerenchyma, thereby changing the environment of the root zone. Thus, if a flooded condition is maintained during the rice growing season, and the soil is dried before harvesting, the redox state of the soil will be changed (Kögel-Knabner et al. 2010). In the oxidized state, the crops absorb ionic Cd^2+^ under the flooded condition because Cd sulfate (CdSO_4_) is a soluble compound. On the other hand, in the reduced state, Cd in the form of Cd sulfide (CdS) precipitates in the soil and cannot be easily transferred to crop plants (Nakanishi et al. 2006). Pinson et al. (2015) confirmed that the Cd content in seeds under flooded conditions was lower than that under non flooded conditions in 1763 rice cultivars.

The arsenic species include both Arsenate (As(V)) and Arsenite (As(III)). As(III) is more toxic than As(V) and has fluid characteristics (Fitz and Wenzel 2002). While As(V) is bound to Fe(III) oxide-hydroxide under non-flooding conditions in soil, As(III), which is soluble under flooded conditions, is easily absorbed into crops by aquaporin channels (Mitani-Ueno et al. 2011). The nudulin 26-like intrinsic proteins (NIP) transporter (OsLsi1), a subfamily of aquaporins, is permeable only to As(III) (Ma et al. 2008; Xu et al. 2015). Thus, in Cd-As polluted soil, the only way to reduce heavy metals in crops is irrigation management.

To find genetic factors affecting Cd uptake and translocation, we performed expression quantitative trait loci (eQTLs) analysis for genome-wide association studies (GWAS) candidate gene and annotated Cd gene. The GWAS is a useful statistical analysis that can confirm genetic variations associated with quantitative traits. However, the functional effects of the candidate genes found in GWAS remain largely unexplained (Altshuler et al. 2008). On the other hand, eQTL analysis is first used in research on transcriptional regulation in budding yeast (Brem et al. 2002) and has been used for GWAS with sequencing studies on diseases. Thereafter, the relationships between genetic variations and gene expression have been widely used in epigenetics, molecular genetics, and proteomics. The advantage of eQTL analysis is to provide data supporting the effects of important trait-related SNPs (*trans* and *cis*) and biological gene expression information related to GWAS results.

In this study, the correlations between Cd and other inorganic components have been analyzed in the roots, stems, leaves, and unpolished grain of nine rice cultivars planted in contaminated soils. Cd interacts with essential trace elements such as Mn, Cu, Fe and Zn in plants. Rahman et al. (2016) reported that Mn reduce Cd in both the roots and stems of rice during a hydroponic culture experiment. Herawati et al. (2000) confirmed that there is a significant correlation between Cd and Cu content in soil. Furthermore, it was reported that significant difference is existed among Fe, Zn, Cu, Mn and Mg in leaves, roots at both heading and ripening stage under Cd stress (Liu et al. 2003a). The absorption mechanisms of Cd and the other inorganic components have been explained by both confirmation of quantitative trait loci and gene identification through natural variation analysis (Clemens et al. 2013). For instance, the natural resistance-associated macrophage protein (NRAMP) family has evolved gradually in all organisms including bacteria, enzymes, plants and animals, and functions to transport Mn^2+^, Zn^2+^, Cu^2+^, Fe^2+^, Cd^2+^, Ni^2+^, Co^2+^, Al^3+^ and protons at the plasma membrane of cells (Nevo and Nelson 2006). In particular, OsNramp5 is a gene that absorbs Mn and Cd from rice roots, and it is known to be involved in the transport of Cd at the exodermis and endodermis (Sasaki et al. 2012). Also, both OsIRT1 and OsIRT2 play an important role in Fe and Cd absorption, and expression of these genes is increased in the root under iron-deficient conditions (Nakanishi et al. 2006; Bughio et al. 2002). Ishimaru et al. (2006) confirmed that OsIRT1 and OsIRT2 are localized at the plasma membrane in vivo. However. regardless of Fe supply, Cd content of OsNramp5 knockout mutant is lower than that of wild-type in both root and shoot. This result indicates that Cd absorption availability of OsIRT1 is neglected by OsNramp5 knockout mutant (Sasaki et al. 2012). Cd absorbed from the root endodermis is again transported to the xylem by P_1B_ -type ATPases known as heavy metal adenosine triphosphatase (HMAs). HMAs are classified into two groups (Cu/Ag and Zn/Co/Cd/Pb) according to their metal-substrates. OsHMA2 is involved in the transport of Zn and Cd at the pericycle of the root, and at the phloem of enlarged and diffuse vascular bundles in the nodes (Yamaji et al. 2013). According to Ueno et al. (2011), OsHMA3 is located in the tonoplast of all root cells, and OsHMA3 plays an important transporter for controlling Cd accumulation in above-ground part.

Lin et al. (2013) confirmed total of 568 genes responded to both Cu and Cd in rice roots by transcriptome analysis. The 530 genes are up-regulated, and 38 genes are down-regulated under Cu and Cd stress conditions. These regulated genes are involved in biological regulation, metabolism, oxidation and reduction, localization, and response to stimulus.

Generally, Cd is known to be interacted with various mineral elements such as Mn, Zn, Cu, and Fe. However, it is difficult to understand the mechanisms of the absorption, transport, and relationship of Cd and other inorganic components due to the influence of environmental factors and the lack of information on biosynthetic processes and gene functions. Therefore, in this study, associations between the expression level of Cd gene and their genotypes, genetic variation in rice cultivars, and correlations between Cd and other inorganic components (Mg, Mn, Fe, Cu, Zn) were investigated in the rice core collection.

## Materials and Methods

### Plant Materials and RNA Extraction

To analyze the association between Cd gene expression and genotype in the rice core collection, Kongju National University’s Conservation Genome Laboratory cultivated 279 rice cultivars in field experiment in 2015, and extracted RNA from their seedlings, 15 days after the heading date (Additional file 2: Table S1 and S2). RNA was extracted using the Total RNA Prep Kit (QIAGEN, DEU) for plant tissue after grinding milky-stage seeds with liquid nitrogen. The extracted RNA was confirmed by electrophoresis in a 0.7% agarose gel and the absorbance was analyzed by ultra violet (UV) spectrophotometry. The RNA concentration was evaluated by a NanoDrop ND-1000 (DuPont Agricultural Genomics Laboratory), and the RNA purity was assessed. The concentrations of the samples were adjusted to 20 ng uL^− 1^, and they were stored in a deep freezer at − 80 °C. A short-read sequence was generated using a HiSeq 2500 (Illumina) to perform next generation DNA sequencing (NGS) for genome reanalysis. The short-read sequences from RNA resequencing were aligned by using the Bowtie and Tophat programs to compare the International Rice Genome Sequencing Project (IRSGP 1.0) and short read sequences (Heo et al. 2017).

### Cd and Other Inorganic Components in Rice

The inorganic components of 279 rice cultivars cultivated in unpolluted soil were analyzed, and nine rice cultivars (RWG-162, RWG-184, RWG-193, RWG-228, RWG-235, RWG-249, RWG-277, RWG-282, and RWG-283) identified in the haplotype and phylogenetic analyses of Os01g0611300 (GWAS candidate gene), were planted in contaminated field located at Yesan-gun Chungcheongnam-do, Republic of Korea. The roots, stems, leaves, unpolished grain, and contaminated soil were analyzed in three replicates. Chemical properties analysis for soil was conducted based on the National Academy Agriculture Science (NAAS 2010). The soil samples were mixed with deionized water in a ratio of 1: 5, stirred for 1 h, and then pH and EC of soil samples were measured by pH / EC meter (Orion 3 star, Thermo, USA). The Cd content in soil based on Korean Soil Environment Conservation Act was analyzed (Minister of Environment, Korea 2010). The 1 mL deionized water, 21 mL HCl, 7 mL HNO_3_ were added to 3 g soil, and then soil was decomposed by using Kjeldahl (C. Gerhardt GmbH & Co., Northants, UK). Decomposed soil samples were filtered using Whatman No. 42 filter paper. In addition, to analyzed the exchangeable cations (Ca, Mg, K, Na), soil samples were shaken with 1 M NH_4_OAc (pH 7.0), and then were filtered by using Whatman No. 42 filter paper (Kang et al. 2018) (Additional file 3: Table S1 and S2).

The fresh weight of samples was measured to analyze the inorganic components in unpolished grain. Also, the samples were dried at 105 °C for two days (48 h), and then they were measured for dry weight. The samples were ground using a cyclone mill (Micro Hammer / Cutter Mill, Switzerland). Then, 0.3 g of each ground sample was weighed and placed in a microwave Teflon vessel. The samples were soaked with a combination of 8 mL HNO_3_ and 1 mL H_2_O_2_ and then digested using a microwave oven (Ethos1, Milestone, USA). The details of the microwave digestion method are described in Table 1. The digested samples were stored at − 20 °C for one hour to reduce the loss of volatile heavy metals through emission of NOx gas and were diluted to 50 ml volumes with deionized water. The samples were then filtered with a 0.45 μm filter to remove the silica. The certified reference materials (IRMM-804 Rice Flour) were also acid decomposed to confirm the recovery rate.

**Table 1:** Operating conditions for microwave digestion

Status	Step	Power (W)	Temp (°C)	Duration (min)
Digestion	1	1000	80	5
	2	1000	100	5
	3	1000	150	5
	4	1000	180	5
	5	1000	180	20

By the method of Marin et al. (1993), the stems and leaves of rice cultivars were washed for 6~7 times with deionized water. The root was sonicated for 3 h to completely remove the soil around root, and then it was rinsed with deionized water and 0.1 *N* HCl for 4~5 times. The analytical pretreatment on root, stem, leaf was same that of unpolished grain.

The Cd content in plant tissue was analyzed in units of μg kg^− 1^ by inductively coupled plasma mass spectrometry (ICP-MS 7700E, Agilent Technologies, USA) and the content of Mg, Mn, Fe, Cu, Zn, and soil were analyzed in units of mg kg^− 1^ using an inductively coupled plasma optical emission spectrometer (ICP-OES Integra XL, GBC, Australia. and ICP-OES 720, Agilent technologies, USA).

### Haplotype and eQTL Analysis of Cd Gene

The gene family such as iron regulated transporter (IRT), NRAMP, HMA, metal tolerance protein (MTP), and low cadmium protein (LCD) is known to be involved in Cd uptake and translocation from roots to stems in rice. Therefore, the expression level of Cd gene in the rice core collection, which were collected from Kongju National University, was analyzed. The eQTL discovery of Cd gene is conducted using efficient mixed model association algorithm (EMMA) and mixed linear model (MLM) in R package. EMMA reduce time when estimating variance components in MLM, and the MLM estimate information and relationship among individuals by random effect (Yu et al. 2006; Kang et al. 2008).

The regulatory factors associated with expression of Cd and GWAS candidate genes have identified in the Rice annotation project database (RAP-DB, http://rapdb.dna.affrc.go.jp/). The difference between the groups of cultivars with different sequences was tested for significance using ANOVA and Duncan’s multiple test in SAS (Statistical Analysis System University Edition).

## Results

### Expression Association Analysis of Cd Gene in the Rice Core Collection

In the eQTL analysis, Cd gene (Os01g0178300 (OsCDT3), Os01g0956700 (OsLCD), Os05g0128400 (OsMTP1), Os06g0102300 (OsPCS), and Os11g0485200) showed significant associations (*p* < 0.05) between gene expression level and genotype. The heritability of OsLCD, OsMTP1 and Os11g0485200 was about 100%, 77%, and 81.5%, respectively, but those of OsCDT3 and OsPCS was approximately 0.7% and 46.1%, respectively in the rice core collection.

### Haplotypes and eQTLs of Os01g0956700 (OsLCD)

**Fig. 1:**
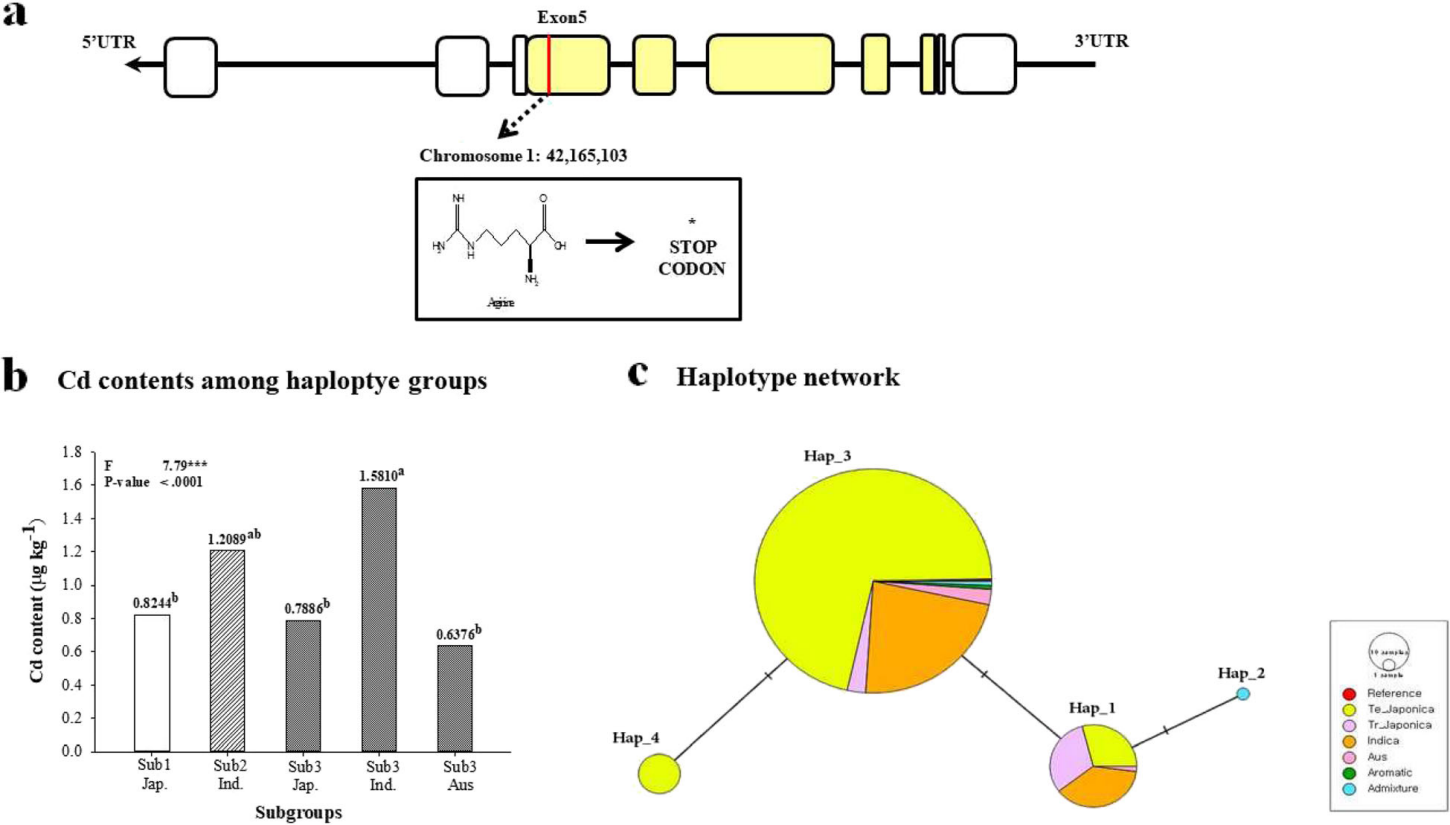
Diversity of rice germplasm for OsLCD. **a** Haplotype analysis including introns and exons of OsLCD. In this figure, yellow represents exon sites; **b** Differences in Cd content among haplotype groups classified by ecotype and genotype; **c** Haplotype network representing the differences in genotype and ecotype among rice cultivars for OsLCD. The ““indicates that there is a difference in one SNP among the groups

Haplotype analyses of exon and intron sites revealed that the genotypes were divided into three groups. Group 3 was divided into three subgroups according to the ecotype: Japonica, Indica, and Aus. There was a significant difference in the Cd content associated with the genotypes at the 42,163,013, 42,165,103, 42,165,481 and 42,165,960 positions between Sub3 (Ind.) and Sub3 (Aus) (Fig. 1).

**Fig. 2:**
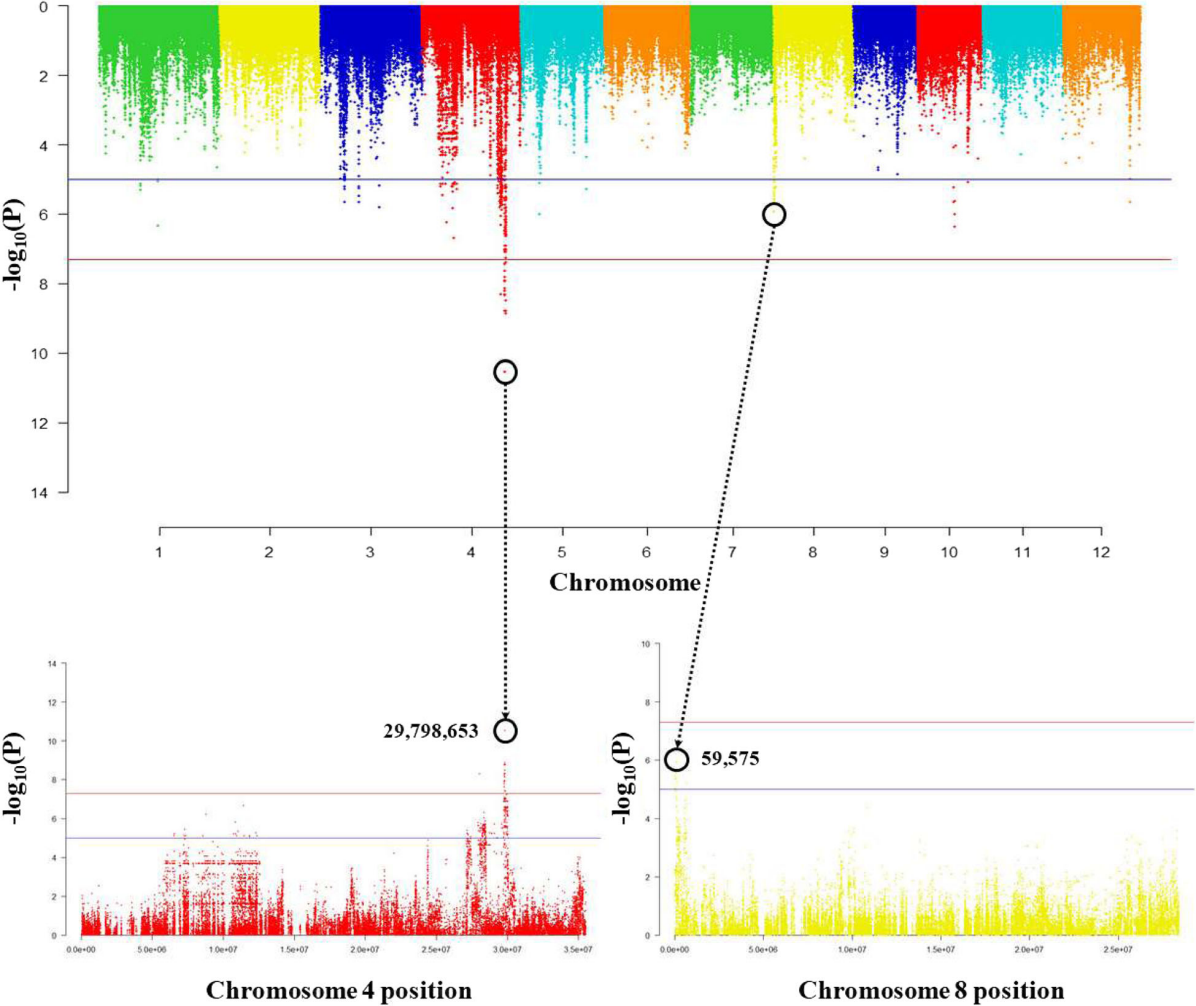
Results of association analysis between genotype and expression level of the annotated Cd gene, OsLCD. The expression quantitative trait loci with strong associations between genotype and expression level are located on chromosomes 4 and 8

In the rice core collection, OsLCD was showed a strong association between genotype and gene expression level both at the SNP position 29,798,653 (−log_10_Pval = 10.53) on chromosome 4 and at the SNP position 59,575 (−log_10_Pval = 5.93) on chromosome 8 (Fig. 2). In other words, OsLCD was more affected by *trans* regulatory factors than *cis*. The 155 *trans*-eQTLs genes were searched within the range of 1 Mb (±500 kb) at the SNP position 29,798,653 on chromosome 4, and the heavy metal associated gene ‘Os04g0590100’ was found. Additionally, the 92 *trans*-eQTLs genes were searched within the range of 1 Mb (±500 kb) at SNP position 59,575 on chromosome 8. Os08g0109200 is associated with S-glutathione dehydrogenase, and Os08g0105600, Os08g0105800, Os08g0106300 are related to cytochrome P450 (Additional file 4: Table S1).

### Haplotypes and eQTLs of Os05g0128400 (OsMTP1)

**Fig. 3:**
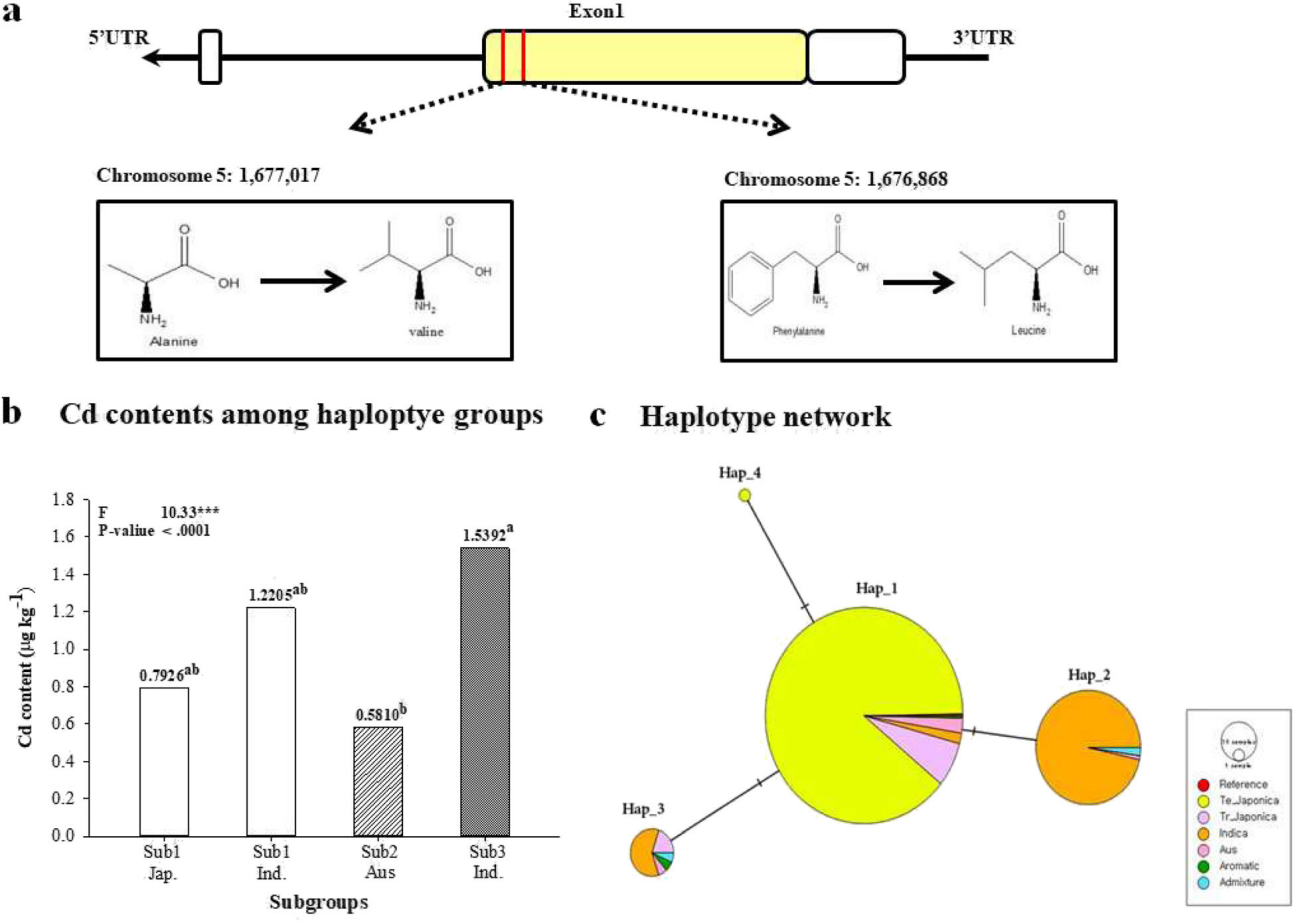
Diversity of rice germplasm for OsMTP1. **a** Haplotype analysis including introns and exons of OsMTP1. Yellow represents exon sites in this figure; **b** Differences in Cd content among haplotype groups classified by ecotype and genotype; **c** Haplotype network representing the differences in genotype and ecotype among rice cultivars for OsMTP1. The “” indicates that there is a difference in one SNP among the groups

In the rice core collection, the genotype of OsMTP1 was divided into four groups. Its genotype was classified based on the 1,676,671, 1,676,737 and 1,676,868 positions which are synonymous SNPs. The genotype was divided into four groups according to ecotype. In these results, Sub2 (Aus.), which is a subgroup of Group 2 had significant differences in Cd content compared to Sub3 (Ind.) of Group 3 (Fig. 3).

**Fig. 4:**
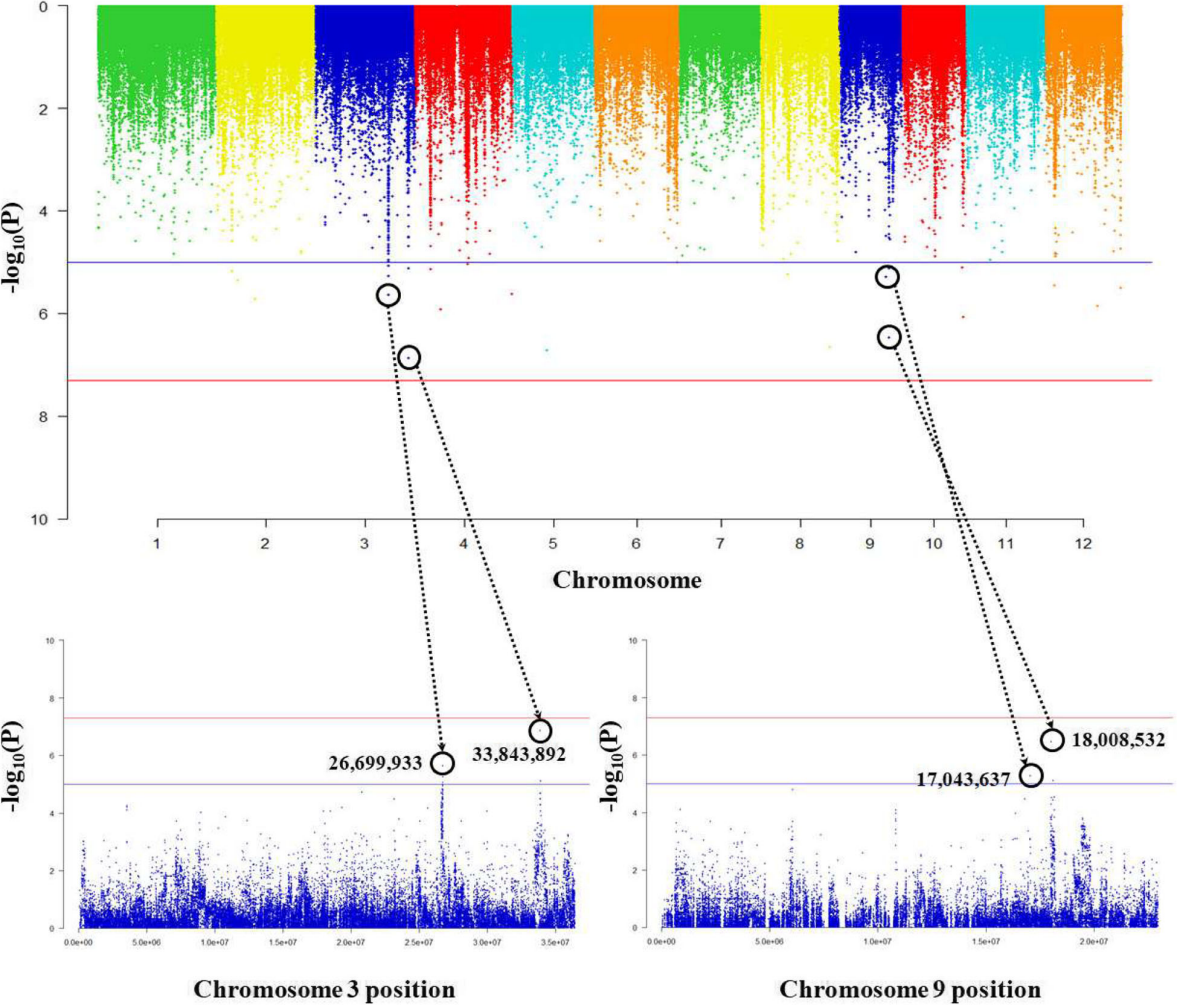
Results of association analysis between genotype and expression level of the annotated Cd gene, OsMTP1. The expression quantitative trait loci with strong associations between genotype and expression level are located on chromosomes 3 and 9

The association between rice genotype and OsMTP1 expression level was analyzed. OsMTP1 was more affected by *trans* than *cis* regulatory factors in the rice core collection. The eQTL analysis showed a strong association between genotype and expression level at the SNP positions 33,843,892 (−log_10_Pval = 6.86) and 26,699,933 (−log_10_Pval = 5.64) on chromosome 3, at the SNP positions 18,008,532 (−log_10_Pval = 6.46) and 17,043,637 (−log_10_Pval = 5.28) on chromosome 9 (Fig. 4). Thus, based on the eQTLs of chromosome 3 and 9, total 539 *trans*-eQTLs genes related to OsMTP1 were detected within 1 Mb (±500 kb) range. Os03g0817300 has a role of a negative regulator on Cd tolerance, Os03g0667300 (OsIRT2) and Os03g0667500 (OsIRT1) have metal transport functions. In addition, EmBP-1-related gene (Os03g0809200; transcription factor), a phytosulfokine-related gene (Os03g0675600) and two growth-regulating factor genes (Os03g0674600 and Os03g0674700) were identified on chromosome 3. On chromosome 9, Os09g0467200 associated with glutathione S-transferase GST 23, and Os09g0454900 involved in serine/threonine protein kinase activity were identified (Additional file 4: Table S2).

### Haplotypes and eQTLs of Os11g0485200

**Fig. 5:**
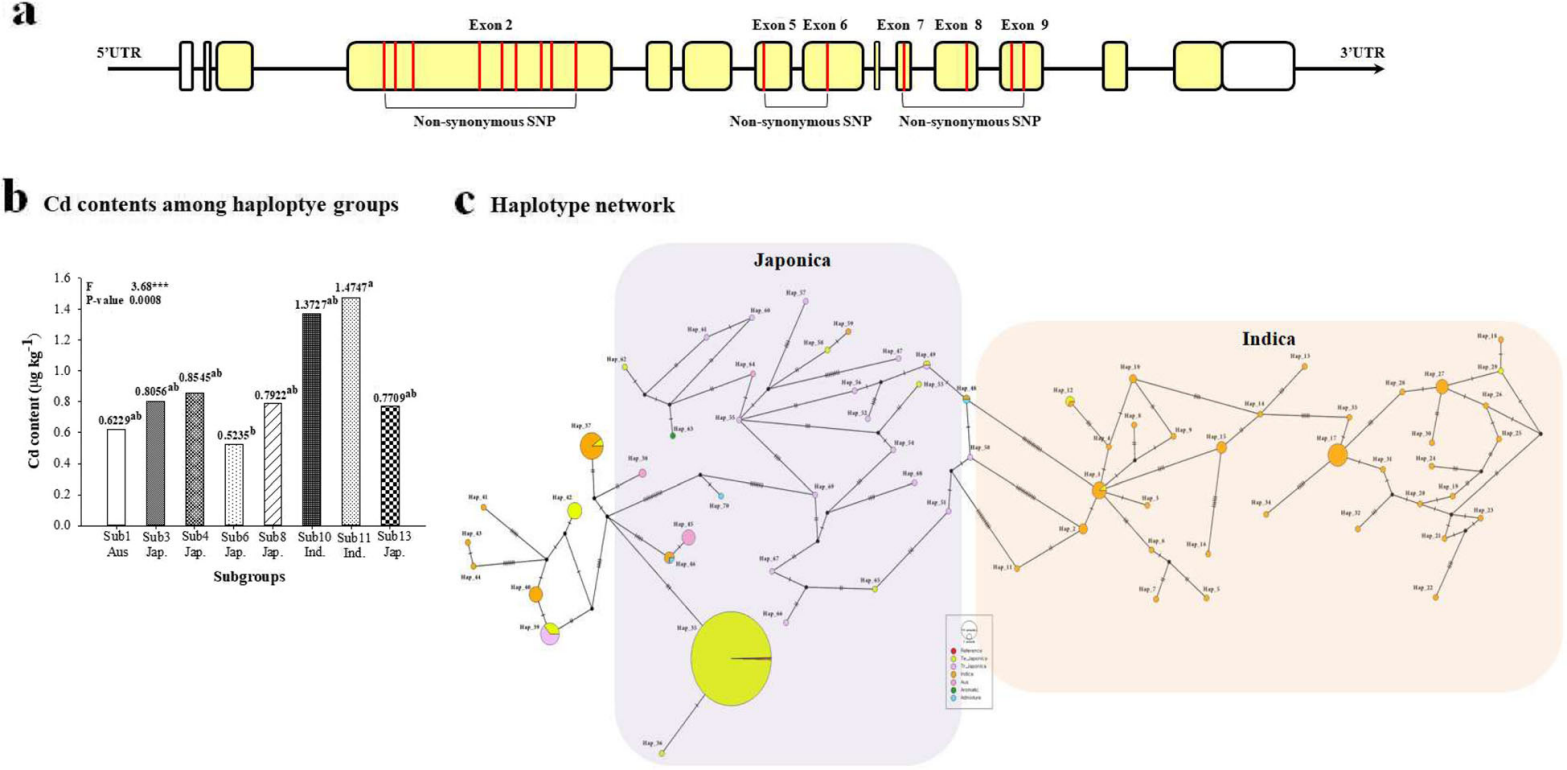
Diversity of rice germplasm for Os11g0485200**. a** Haplotype analysis including introns and exons of Os11g0485200; **b** Differences in Cd content among haplotype groups classified by ecotype and genotype; **c** Haplotype network representing the differences in genotype and ecotype among rice cultivars for Os11g0485200. ““or ““indicates that there is a difference in SNPs among the groups

The genotype of Os11g0485200 in the rice core collection was divided into 13 groups. In the haplotype results, there was a significant difference in the Cd content by genotype between Group11 (Sub11 Ind.) and Group 6 (Sub6 Jap.). Group6 (Sub6 Jap.) had nonsynonymous SNPs (5′-T**A**CCAAGCG-**G**-GTCG-**G**-T-**T**T-3′) in the third, sixth, ninth, and eleventh exons, while Group 11 (Sub11 Ind.) had nonsynonymous SNPs (5′-**C**G**TTGGATA**-C-**A**TCG-A-**C**-A**A**) in the third, seventh, tenth and eleventh exons (Fig. 5).

**Fig. 6:**
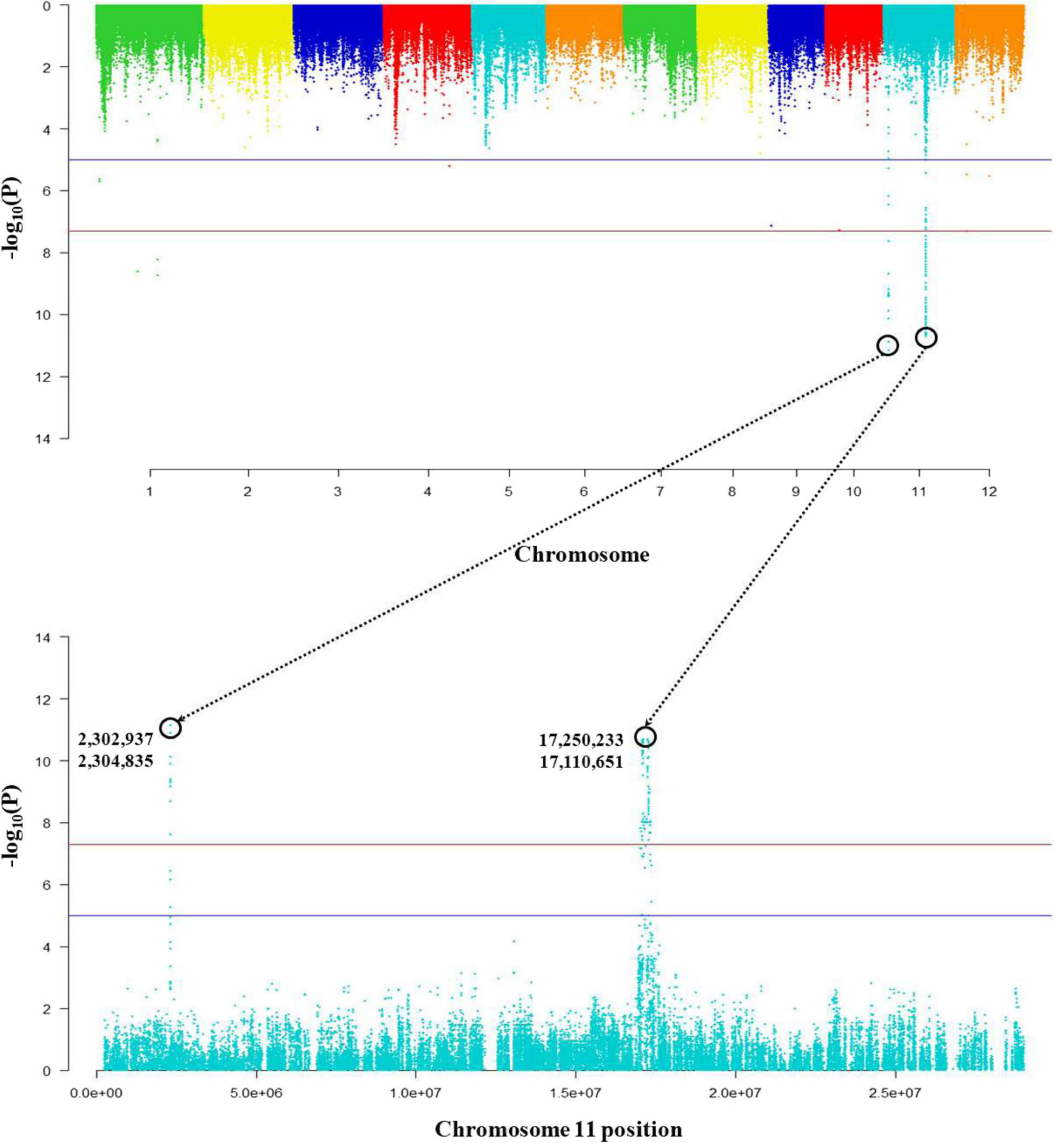
Os11g0485200 is associated with *cis* regulatory factors. The expression quantitative trait loci with strong associations between genotype and expression level are located on chromosome 11

The expression level of Os11g0485200 was most associated with genotypes on chromosome 11. The *P*-values (−log_10_Pval) of the SNP positions at 2,302,937, 2,304,835, 17,110,651 and 17,250,233 were 11.16, 10.89, 10.71, and 10.71, respectively. It was determined that Os11g0485200 was more affected by *cis* regulatory elements than *trans* (Fig. 6). The 225 *cis*-eQTLs genes were detected on chromosome 11, of which Os11g0147500 has heavy metal transport and detoxification functions. In addition, genes (Os11g0484400, Os11g0484500, Os11g0484700, Os11g0488500, Os11g0489250 and Os11g0490200) which involved in redox, inhibition of lipid transport and seed storage were detected within 1 Mb (±500 kb) range (Additional file 4: Table S3).

### Candidate Cd Gene from GWAS and its eQTLs

**Fig. 7:**
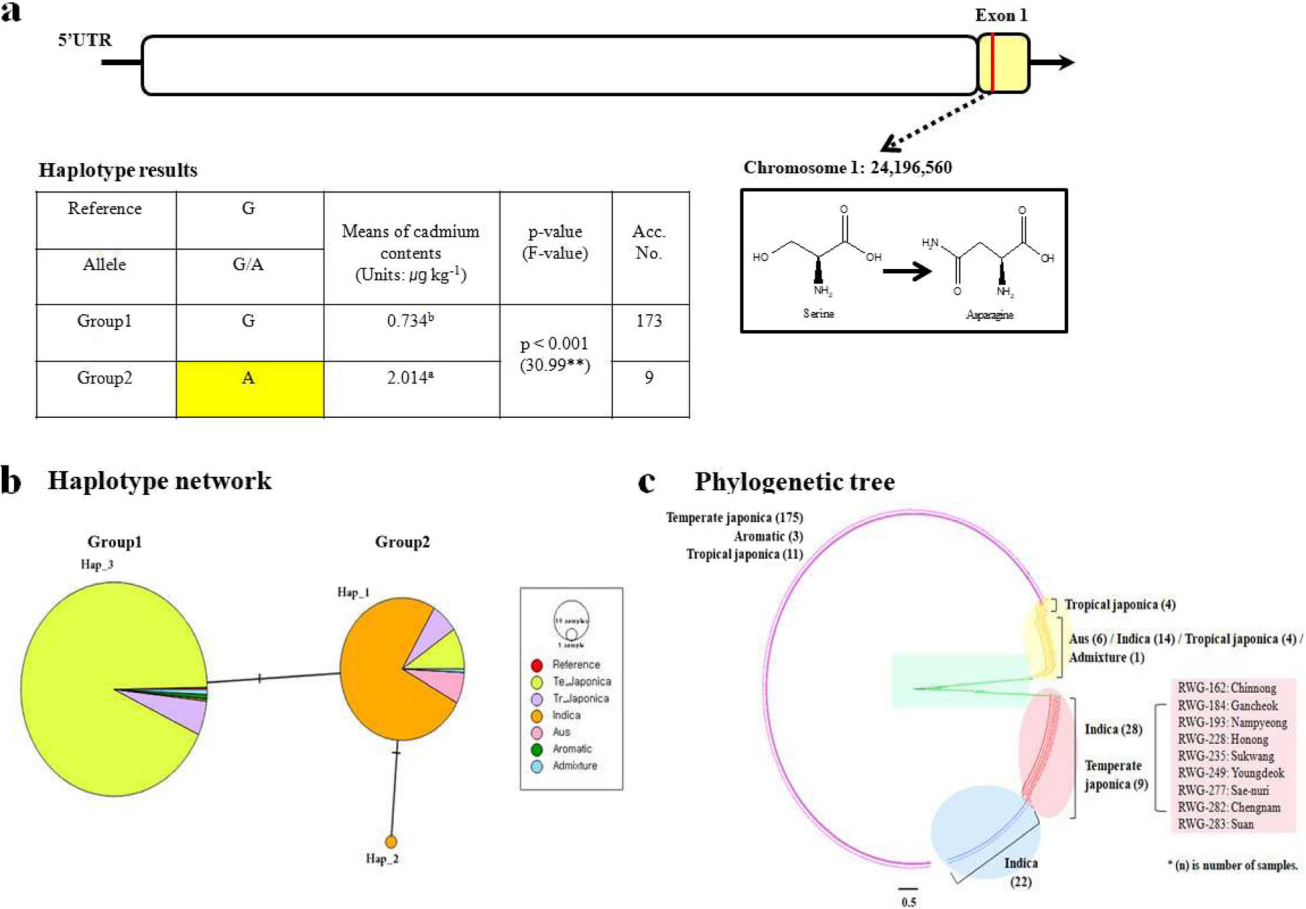
Genome-wide association studies (GWAS) for candidate genes for Cd content in 182 temperate Japonica (*Oryza sativa*) cultivars. **a** Results of haplotype analysis; **b**, **c** Results of haplotype network and phylogenetic tree

In previous study, GWAS was conducted for Cd content in the unpolished grain of 182 temperate Japonica rice (*Oryza. sativa*) cultivars. As a result, Os01g0611300, a candidate gene associated with metal tolerance within the range of ±25 kb a SNP position with a *P*-value of 6.03, was identified. The genotype of Os01g0611300 has divided into two groups, wherein Group 2 had a nonsynonymous SNP (Serine → Asparagine) at 24,196,560 position on exon. The Cd content (2.014 μg kg^− 1^) of Group 2 was significantly higher (*p* < 0.05) than that of Group 1 (0.734 μg kg^− 1^). As shown in the phylogenetic tree, the nine cultivars (RWG-162, RWG-184, RWG-193, RWG-228, RWG-235, RWG-249, RWG-277, RWG-282 and RWG-283) belonging to Group 2 have been closer to Indica than Japonica (Fig. 7) (Lee et al. 2016). Therefore, field experiment was carried out on these nine rice cultivars to confirm Cd uptake and translocation.

**Fig. 8:**
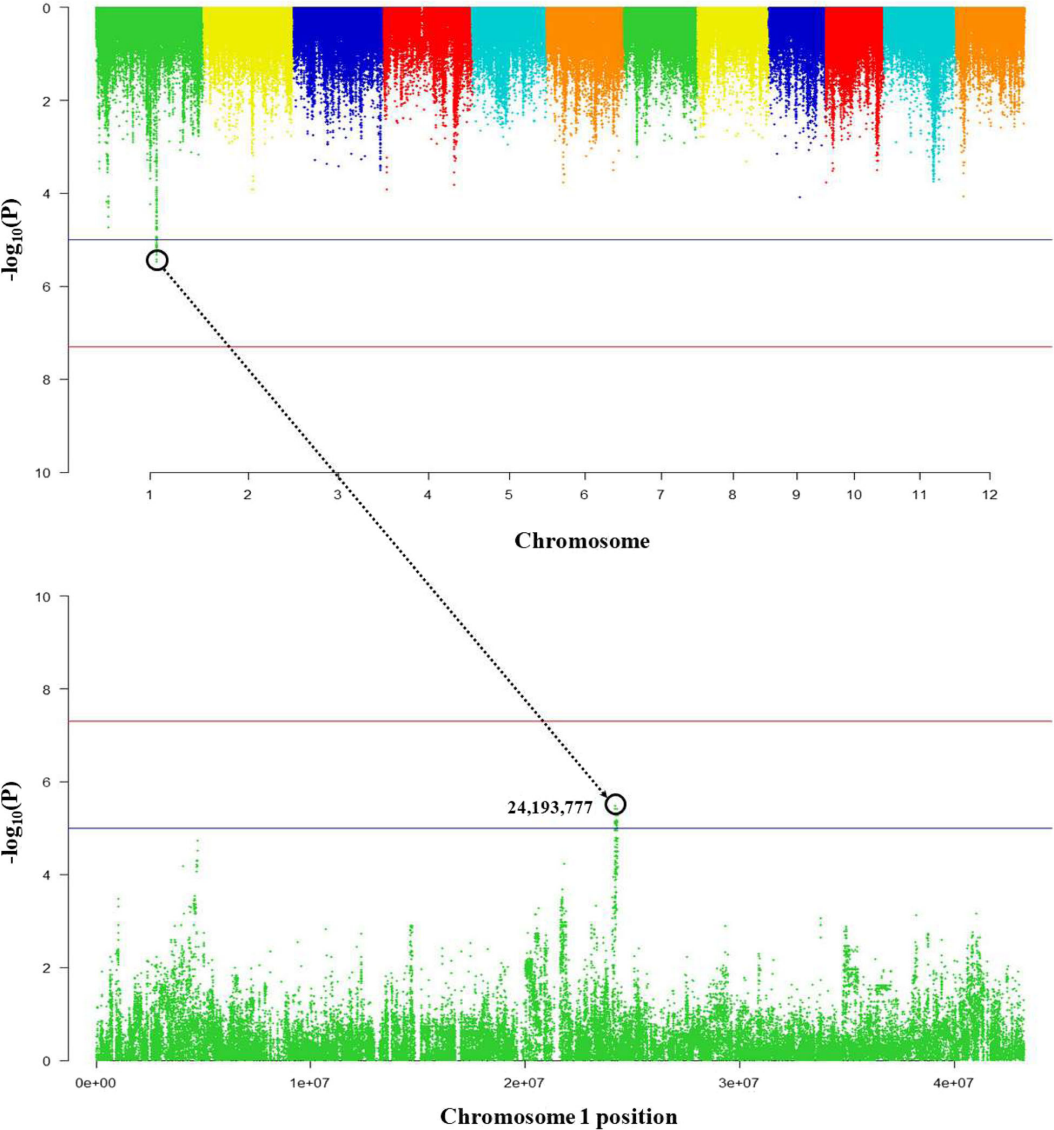
Os01g0611300 is associated with *cis* regulatory factors. The expression quantitative trait loci with strong associations between genotype and expression level are located on chromosome 1

According to the eQTL analysis, Os01g0611300 expression was more affected by *cis* regulatory factors than *trans.* The 24,193,777 SNP position had the highest *P*-value (−log_10_Pval) of 5.47 on chromosome 1, and the 127 *cis-*eQTLs genes were identified within a range of 1 Mb (±500 kb) based on eQTLs. Among the *cis*-eQTLs genes, only Os01g0611900 is a pentatricopeptide repeat-containing (PPR) protein, and Os01g0610800 is a protein associated with thrombospondin type 1 repeats. In addition, there are genes related to metabolic processes, cellular components, DNA binding, and zinc ion binding within *cis* regions (Fig. 8) (Additional file 4: Table S4).

### Filed Experiment on Contaminated Paddy Soil

The analysis of Cd content in the unpolished grain revealed various differences among the nine rice cultivars. The Cd content of RWG-162, RWG-193, and RWG-235 were low at 0.0024, 0.0029, and 0.0031 mg kg^− 1^, respectively. On the other hand, the cultivars with high Cd content were RWG-282 (0.0056 mg kg^− 1^), RWG-184 (0.0055 mg kg^− 1^) and RWG-228 (0.0053 mg kg^− 1^).

There was a statistically significant difference between the cultivars with high Cd content and the cultivars with low content. The Cd content of RWG-277 was significantly different from those of RWG-184 and RWG-282. However, the Cd content of RWG-249 and RWG-283 were not significantly different from those of the other cultivars.

**Fig. 9:**
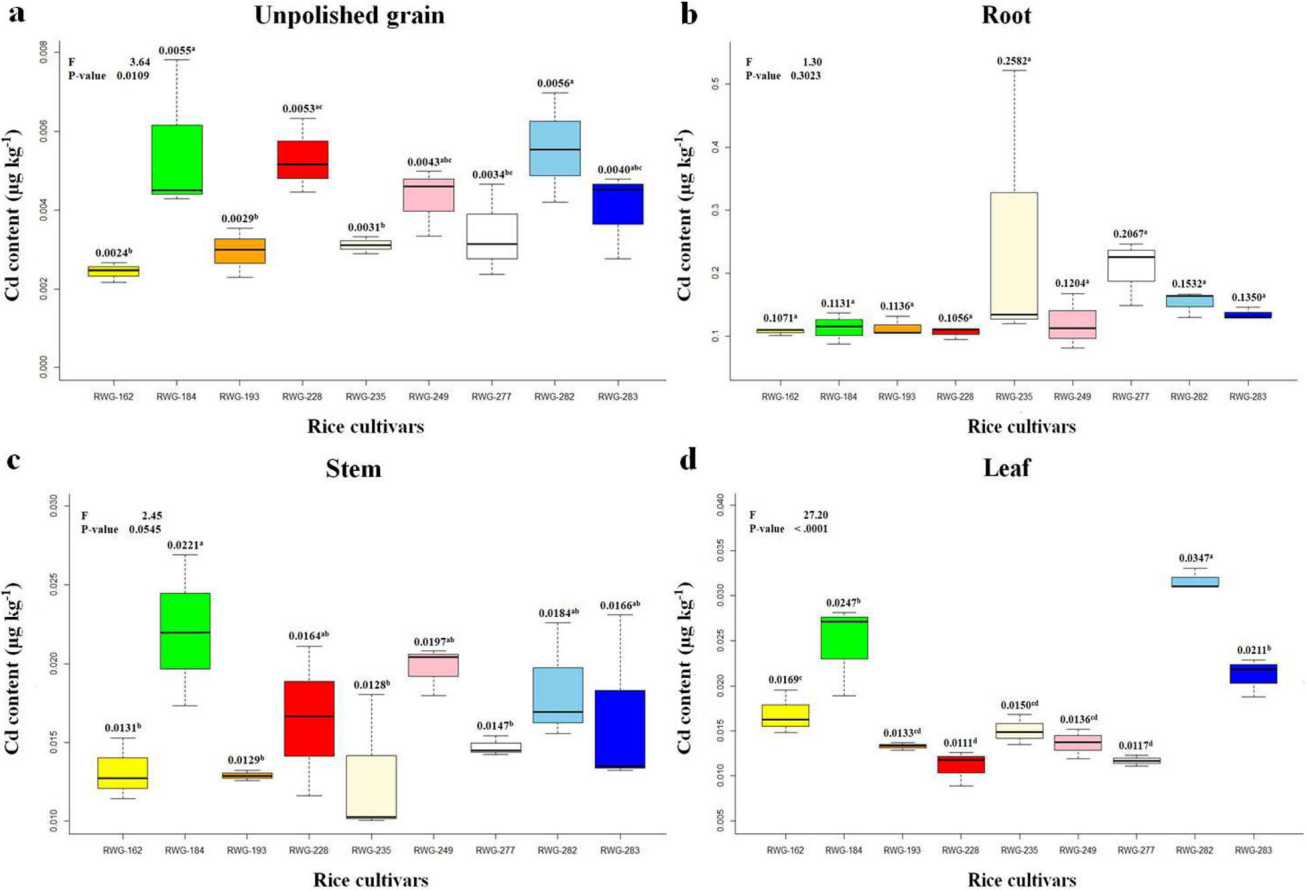
The Cd content in unpolished grain, leaves, and stems was different among rice cultivars at p <0.01 and 0.05 levels. However, the Cd content in the roots was not significantly different among cultivars. The numbers above each boxplot indicate the average Cd content. **a** The Cd content in unpolished grain; **b** The Cd content in root; **c** The Cd content in stem; **d** The Cd content in leaf

There was no difference in Cd in roots, but there was a significant difference in Cd in stems and leaves. The Cd content in the stems of RWG-184 was 0.0221 mg kg^− 1^, which was higher than those of RWG-162, RWG-193, RWG-235 and RWG-277. However, there was no statistically significant difference in Cd content in stems among RWG-184, RWG-228, RWG-249, RWG-282 and RWG-283. For leaves, the highest Cd content, in RWG-282 was 0.0317 mg kg^− 1^, while RWG-193, RWG-228, RWG-235, RWG-249 and RWG277 had low Cd content relatively. The Cd content in the leaves of RWG-184 and RWG-283 were 0.0247 and 0.0211 mg kg^− 1^, respectively. RWG-184 showed higher Cd content in the leaves, stems and unpolished grain than the other rice cultivars, but had low Cd in the roots. (Fig. 9).

There were positive correlations between Cu and Zn in the roots and unpolished grain, respectively. In stems, there were positive correlations between Mg and Fe and between Cu and Cd. Additionally, Mg and Mn as well as Zn and Mn had significant positive correlations in the leaves.

## Discussion

### Genome and Transcriptome Analyses of Cd Gene

In this study, it analyzed whether the genotypes of Cd gene are associated with gene expression level in the rice core collection or not. The Cd transporter (OsLCD, OsMTP1 and Os11g0485200) and the tolerance proteins (OsCDT3 and OsPCS) were showed strong association between expression level and genotypes.

OsPCS is a gene encoding phytochelatin synthases (PCS) and is involved in the accumulation of Cd and As in rice seeds. Concerning OsPCS, it was reported that rice has two PCS genes in genome on chromosome 5 (OsPCS1) and 6 (OsPCS2). OsPCS1 is more activated by As (III) than Cd, while OsPCS2 is more activated by Cd than As (III) (Yamazaki et al. 2017). In addition, Hayashi et al. (2017) confirmed that OsPCS1 is saturated at lower As concentration than OsPCS2 in PC synthesis assay. These data suggest that OsPCS1 may contribute to regulation of As (III) level, whereas OsPCS2 is involved in sequestration of As and may contribute to Cd detoxification in rice.

OsCDT3 is a protein that involved in tolerance of Cd and Al at plasma membrane. However, knockdown of OsCDT3 in yeast and RNAi experiment increase sensitivity to Al toxicity, but does not affect the tolerance to Cd toxicity (Xia et al. 2013).

In this study, OsCDT3 and OsPCS heritability (respectively 0.7 and 46.1%) was less than 50% in the rice core collection. By compressed mixed linear model, homogeneous variance is assumed for the residual effect. σ^2^_a_ means the genetic variance and σ^2^_e_ means the residual variance. In other words, the proportion of total variation (σ^2^_a_ + σ^2^_e_) explained by genetic variation (σ^2^_a_) is defined as heritability (h_2_) (Zhang et al. 2010). This result indicates that these genes is more affected by residual variance than by genetic variance in rice core collection. Therefore, this study is analyzed the sequences of the Cd transporter genes (OsLCD, OsMTP1, and Os11g0485200) with more than 50% heritability and strong associations between genotype and gene expression level.

In haplotype analysis, the genotype group was subdivided according to ecotype (Japonica, Indica and Aus). Furthermore, rice cultivars with a small number of ecotypes in each group were excluded from the statistical analysis because the sample size is too small to represent the population.

OsLCD has important functions related to Cd tolerance and accumulation. OsLCD is located in the cytoplasm and nucleus. In particular, this gene is observed in the root vascular bundle and in the phloem companion cells of leaves. It plays an important role in the transport and accumulation of Cd in plants (Shimo et al. 2011). Thus, haplotype analysis was conducted for the exon and intron sites; the genotype of OsLCD was classified into three groups, and there was no significant difference among the genotypes with respect to Cd content. However, there was a significant difference between the Cd content of Indica and Aus. within the same genotype group. Therefore, Cd content seem to be influenced by ecotype than genotype of rice cultivars in OsLCD.

MTPs are cation diffusion facilitator (CDF) proteins that are widely distributed in bacteria, fungi, animals and plants. These proteins are associated with resistance to Co and Mn as well as Ni, Cd, and Zn. According to the protein subcellular localization predictions of WoLFPSORT, OsMTP1 is presumed to be located in the epidermal cell plasma membrane rather than the vacuoles and vesicles (Yuan et al. 2012).

The genotypes of OsMTP1 were classified into three groups, there was no significant difference in Cd content between Japonica and Indica with same genotype. However, there was a significant difference (*p* < 0.0001) in Cd content between Indica and Aus. with different genotypes. This means that the Cd content depends on the genotype and ecotype of the rice cultivars in OsMTP1.

**Fig. 10:**
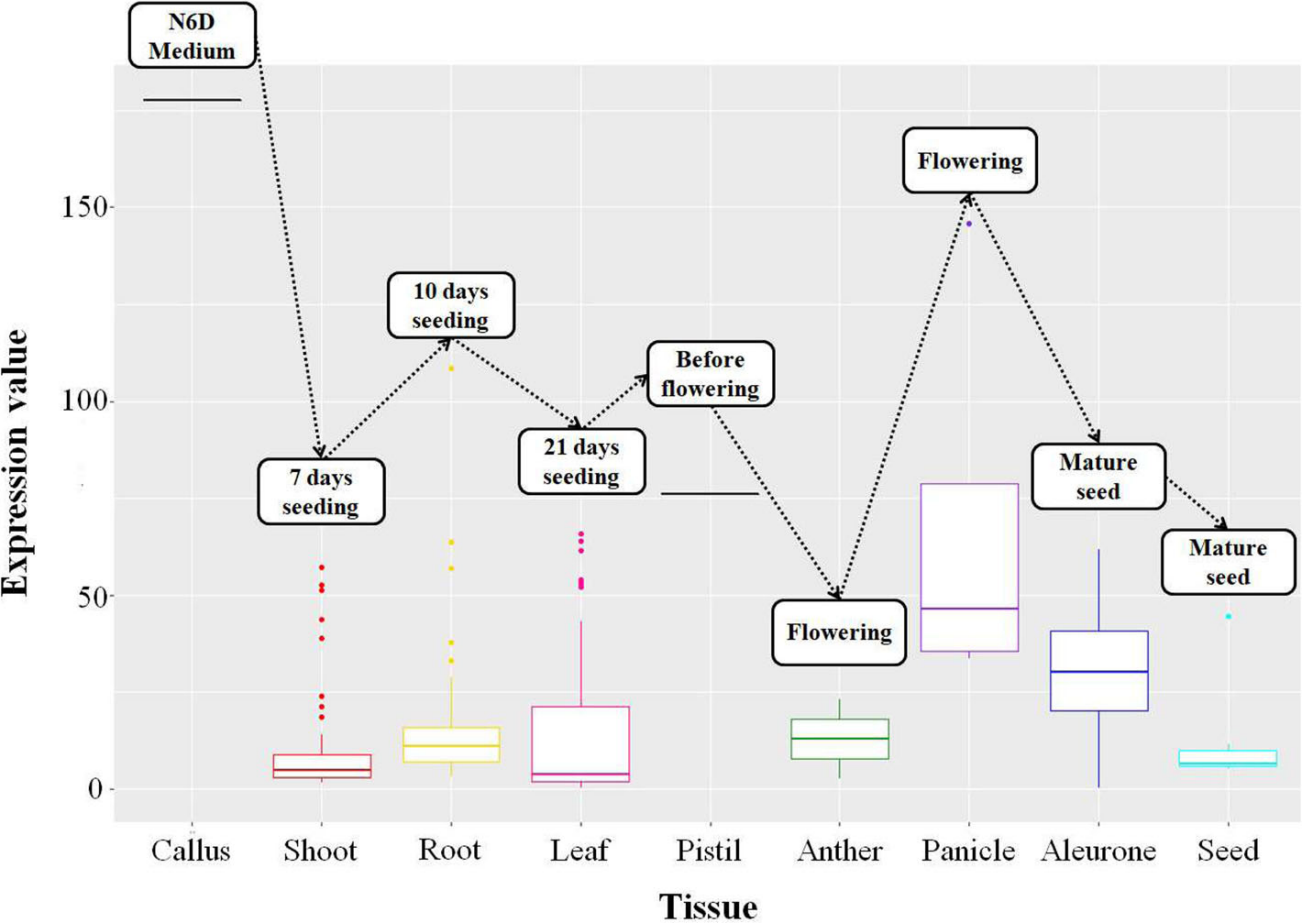
Expression level of Os11g04852200 in each tissue during the growth period of Nipponbare

Os11g0485200 encodes a protein that plays a role in the transport of K, Mg, Cd, Zn, Na, Ca, and H, but little is known about this gene as yet. The expression level of Os11g0485200 is high in callus, flowering panicle, and root at 10 days after sowing during the growth period of Nipponbare based on the genome information of IC4R (Xia et al. 2017; available at http://ic4r.org/) (Fig. 10).

The genotypes of Os11g0485200 were classified thirteen groups, and again subdivided by ecotype. It was confirmed that there was a significant difference (*p* < 0.0008) in Cd content between Japonica and Indica with different genotype.

As shown in above results, the Cd content by specific genotype or ecotype was different. In addition, Cd content of Indica was higher than that of Japonica in Cd gene (OsLCD, OsMTP1, and Os11g0485200). This result is consistent with previous studies (Morishita et al. 1987; Liu et al. 2007).

The definition of *cis* and *trans* is depend on the purpose or subject of study. In study on analysis and visualization tool for eQTLs, the window size of the gene of interest / SNP is adjusted to 2 Mb (Yang et al. 2010). The study on cells and tissues of human defined that polymorphisms within 1 Mb are *cis* for genomic loci related to mRNA expression of specific genes (Stranger et al. 2012). In addition, Cheng et al. (2015) have mapped *cis*-eQTLs within 200 kb by correlating gene expression to genotype in human disease study. On the other hand, Wang et al. (2010) divide the whole genome of rice into 1-cM partitions, and investigate the distribution of eQTLs along with the genome. Therefore, in this study, polymorphisms within 1 Mb are defined as *cis*, and regulatory factors (*cis*-eQTLs and *trans*-eQTLs) have been described mainly on the gene encoding protein. The *cis* and *Trans* genes are identified by RAP-DB. However, regulatory factors include both coding and non-coding regions. Therefore, these regulatory factors associated the expression of target genes need to be validated by further studies.

In this study, not only most *cis*-eQTLs but also most *trans*-eQTLs genes are related to plant stress, metabolites (cytochrome P450 and glutathione, phytosulfokines and serine-threonine protein kinase), transcription factors, growth factors or redox enzymes.

Os04g0590100 is a heavy metal related domain (HMA domain), and it has been detected as a *trans*-eQTLs gene associated with the expression of OsLCD. However, there is no studies on Os04g0590100 to date, only its function has been inferred by the orthologue (At3g04900) of *Arabidopsis thaliana* in EnsemblPlants (n.d.) database (http://plants.ensembl.org). Vaid et al. (2012) classified At3g04900, which is heavy metal-associated isoprenylated plant protein 42 (HIPP 42) with L-type lectin receptor-like kinases (LecRLKs) on the basis of expression profile and phylogenies. LecRLKs are membrane proteins involved in a variety of function from plant growth and development to stress tolerance. In Arabidopsis, L-type LecRLKs genes are expressed about 46.3% in biotic stress condition and about 12.2% in Cd stress condition. More research is needed in the future because the response to individual genes associated with LecRLK is currently unknown under particular stress.

OsIRT1, OsIRT2 and Os03g0817300 are detected as *trans*-eQTLs of OsMTP1. To date OsIRT1 and OsIRT2 are known as transporter proteins of Cd and Fe, but we carefully suggest that OsIRT1 and OsIRT2 act as regulatory factors associated with OsMTP1 expression. Also, Os03g0817300 is only known to act as a negative regulator of Cd tolerance, it may affect the expression of OsMTP1 as a regulatory factor.

The expression of Os11g0485200 is investigated to be more associated with *cis*-eQTLs than *trans*-eQTLs. Os11g0147500 which is heavy metal transport and translation domain was detected in *cis*-eQTLs, but there is no more detailed research until now. Only, At3g05220 with similar function to Os11g0147500 is identified in *Arabidopsis thaliana* by ortholog work in EnsemblPlants (n.d.) database. At3g05220 is known as heavy metal associated isoprenylated plant protein (HIPP34) which is a metallochaperone containing heavy metal binding domains (HMA) and C-terminal isoprenylated motifs, and it safely transports metal ions in cells (Abreu-Neto et al. 2013).

According to Lee et al. (2016), GWAS analysis was performed for 182 temperate Japonica cultivars to identify new genes in addition to the Cd gene already known in rice. From the GWAS results, Os01g0611300 (metal tolerance protein), which is presumed to affect Cd content in rice, was found (Lee et al. 2016). The size of Os01g0611300 is relatively small 1113 bp compared to annotated Cd gene, but it is known as a metal tolerance protein. The genetic variance among rice cultivars in Os01g0611300 was identified by haplotype analysis. The cultivars with higher Cd content in unpolished grain showed significant difference in genotype and phenotype compared to other cultivars. And it found that ecotype of these cultivars with high Cd content was Japonica, but their genotypes were close to Indica in phylogeny analysis. Additionally, the regulatory factors associated with expression of Os01g0611300 were analyzed using eQTL. Os01g0611300 was more affected by *cis*-eQTLs factors than *trans*-eQTLs. The *cis*-eQTLs genes are associated with so many metabolic pathways, DNA - zinc ion binding, thrombospondin and pentatricopeptide *etc*. Thrombospondin is a motif found in animals that plays an important role as a regulator of cellular interactions in vertebrates. It is known to bind growth factors, cytokines, proteases and multiple matrix components (Adams 2001). Also, pentatricopeptide is known to be expressed mainly in plant leaves before flowering under abiotic stress (Ahsan et al. 2007).

Generally, plants synthesize metal-binding peptides such as cys-rich phytochelatins and metallothioneins, in vivo depending on the level of toxicity when exposed to heavy metals, thereby changing gene expression (Jonak et al. 2004). According to a study by Suzuki et al. (2001), mitogen-activated protein kinase (MAPKKK) is among the genes activated during Cd and Cu response in Arabidopsis. In the Arabidopsis genome sequence, 20 mitogen-activated protein kinases (MAPKs), 10 mitogen-activated protein kinase kinases (MAPKKs), and 80 MAPKKKs are identified (Jonak et al. 2002), but the MAPK gene family and its regulatory functions are not well-known in rice (Reyna and Yang 2006). Thereafter, 16 MAPKs and 8 MAPKKs have been reported (Hamel et al. 2006). In addition, 75 MAPKKKs, which are related to plant cytokinesis, ethylene signaling, tolerance, reaction mechanisms and various stress factors, are confirmed in rice by in silico analysis (Rao et al. 2010). When rice is exposed to heavy metals, MAPKKKs, MAPKKs, and MAPKs, which are involved in phosphorylation reactions, are induced (Jonak et al. 2004). In particular, MAPK has been reported to be more activated in Cd-tolerant cultivars than Cd-sensitive cultivars (Yeh et al. 2007). These MAPK signaling pathways are closely related to GSH synthesis (Guan et al. 2016). Limon-Pacheco confirmed that MAPK activity is dependent on the oxidation-reduction reaction caused by GSH decreases in the brain, kidney, and liver of rats (Limon-Pacheco et al. 2007). However, Cd has not been directly observed in the redox reactions of cells, and it is unknown how Cd is involved in the activity of MAPKs such as salt stress-induced MAPK (SIMK), mitogen-activated protein kinase homolog (MMK2), mitogen-activated protein kinase (MMK3) and serine/threonine-protein kinase (SAMK) (Jonak et al. 2004).

In this study, proteins related to GSH synthesis (Phytozulfokin) and MAPK activity metabolites (serine-threonine protein kinase) were detected along with heavy metal-associated proteins within *cis* and *trans*-eQTLs. These *cis* and *trans*-eQTLs genes were closely related to the expression of Cd genes and GWAS candidate gene. Therefore, further studies are required for non-coding regions including proteins encoded as regulatory factors affecting expression of Cd genes or candidate genes.

### Inorganic Components Analysis

The interactions between Cd and other inorganic components are a topic of ongoing study. Cd is closely connected to the absorption and translocation of inorganic components such as Mg, Mn, Fe, Cu, and Zn in plants (Cataldo et al. 1983). Arao and Ishikawa (2006) demonstrated that Cd in stem of rice planted in Cd- polluted paddy soil (1.7 mg kg^− 1^, 2.9 mg kg^− 1^) has a significantly positive correlation with Zn and Mn. Liu et al. (2003b) confirmed the significantly positive correlation among Fe, Zn, Cu, and Mn in the roots at the heading stage, and confirmed the significantly negative correlation between Cd and Mn in the leaves. Moreover, significantly positive correlations are identified among Cd, Fe, Zn, Cu, and Mn in the roots at the ripening stage, as well as significantly positive correlations among Cd, Mg, Fe, Zn, and Cu in the leaves. Shimo et al. (2011) reported that because Cd and Zn have similar physical and chemical properties, they compete for a biological ligand and Cd disturbs the accumulation of Zn. Therefore, when the crop is exposed to Cd, Zn content is reduced in the roots and stems.

Similarly, the Cd gene is involved not only in Cd absorption and transport but also that of cations such as Mg, Mn, Cu, Fe and Zn. In particularly, OsMTP1, OsNramp, and OsIRT families are involved in the absorption of not only Cd but also metal ions, including Zn, Mn, and Fe (Nakanishi et al. 2006; Lee and An 2009; Sasaki et al. 2012; Yuan et al. 2012). OSHMA2 absorbs Cd and Zn from the root xylem, and transports them to the stem phloem (Uraguchi et al. 2011). Takahashi et al. (2012) confirmed that absorption of Cd and Zn is decreased when OsHMA2 expression was inhibited.

When Sasaki et al. (2012) knocked down OsNramp5 which transports Mn, Fe, and Cd in the endodermis and exodermis of roots, Cd, Mn, and Fe are decreased in the roots, stems, and unpolished grain. In contrast, Ishimaru et al. (2012) showed that as the expression of OsNRAMP5 is decreased, Mn is decreased in roots and stems, while Cd is increased in stems. Thus, the interaction between Cd and other inorganic components is not yet clear.

In this study, Cd, Mg, Mn, Cu, Fe and Zn were analyzed in the roots, stems, leaves, and unpolished grain of nine temperate Japonica cultivars close to Indica in the phylogenetic analysis of the metal tolerance protein, Os01g0611300.

RWG-184 showed higher Cd content in leaves, stems and unpolished grain than the other rice cultivars, and lower Cd content in roots. This result means that RWG-184 absorbed and transported more Cd from the root to the shoot than the other rice cultivars. On the other hand, RWG-235 had high Cd content in roots but low content in leaves, stems, and seeds. In other words, RWG-235 transported less Cd from root to shoot, unlike RWG184. Similarly, Liu et al. (2003a) reported that Cd concentration ratios in roots, stems, and leaves are different for each cultivar based on data collected over 2 years.

The correlation among inorganic components in roots, stems, leaves and unpolished grain of nine rice cultivars was also analyzed. As a result, it was confirmed that there was a positive correlation between the inorganic components. There was a positive correlation between Cd and Cu content in the stem, and between Mn and Mg content in the leaves. However, RWG-162 and RWG-184 were higher Cu content in stems than the other cultivars, but RWG-162 was low Cd content in the stem. In addition, RWG-228 was low Mn content in the leaf, but the Mg content of RWG-228 was not lower than that of other cultivars. Therefore, this result suggests that the correlation among inorganic components is may different depending on the inherent characteristics of the rice cultivars.

## Supplementary information

**Additional file 1: Figure S1.** Glutathione (GSH) biosynthetic process for Cd uptake in plants. Amplitude-phase shift keying (APSK) and phosphoadenosine phosphosulfate reductase (PAPSR) involved in GSH synthesis. **a** Sulfate assimilation pathway and biosynthetic process of cysteine; **b** GSH synthesis and ROS processing. *This figure is a reconstruction of the original source (Mendoza-Cozatl et al. 2005).

**Additional file 2: Table S1.** DNA sequencing and rice core collection information for 279 cultivars. **Table S2.** RNA sequencing information for 163 cultivars.

**Additional file 3: Table S1.** The inorganic component concentration in contaminated paddy soils. **Table S2** Chemical properties in the contaminated paddy soils.

**Additional file 4: Table S1.** Proteins associated with expression of OsLCD by eQTL analysis. **Table S2.** Proteins associated with expression of OsMTP1 by eQTL analysis. **Table S3.** Proteins associated with expression of Os11g0485200 by eQTL analysis. **Table S4.** Proteins associated with expression of GWAS candidate gene (Os01g0611300) by eQTL analysis.

## Data Availability

The data that support the findings of this study are available from the corresponding author upon reasonable request.

## References

[CR1] Abreu-Neto JB, Turchetto-Zolet AC, Oliveira LFV, Helena M, Zanettini B, Margis-Pinheiro M (2013). Heavy metal-associated isoprenylated plant protein (HIPP): characterization of a family of proteins exclusive to plants. FEBS J.

[CR2] Adams JC (2001). Thrombospondins: multifunctional regulators of cell interactions. Annu Rev Cell Dev Biol.

[CR3] Ahsan N, Lee SH, Lee DG, Lee H, Lee SW, Bahk JD, Lee BH (2007). Physiological and protein profiles alternation of germinating rice seedlings exposed to acute cadmium toxicity. C R Biol.

[CR4] Altshuler D, Daly MJ, Lander ES (2008). Genetic mapping in human disease. Science..

[CR5] Arao T, Ishikawa S (2006). Genotypic differences in cadmium concentration and distribution of soybean and rice. Japan Agric Res Q.

[CR6] Brem RB, Yvert G, Clinton R, Kruglyak L (2002). Genetic dissection of transcriptional regulation in budding yeast. Science.

[CR7] Bughio N, Yamaguchi H, Nishizawa NK, Nakanishi H, Mori S (2002). Cloning an iron-regulated metal transporter from rice. J Exp Bot.

[CR8] Cataldo DA, Garland TR, Wildung RE (1983). Cadmium uptake kinetics in intact soybean plants. Plant Physiol.

[CR9] Cheng Z, Chu H, Fan Y, Li C, Song YQ, Zhou J, Yuen KY (2015). PExFInS: An integrative post-GWAS explorer for functional Indels and SNPs. Scientific Reports.

[CR10] Clemens S (2001). Molecular mechanisms of plant metal tolerance and homeostasis. Planta.

[CR11] Clemens S, Aarts MG, Thomine S, Verbruggen N (2013). Plant science: the key to preventing slow cadmium poisoning. Trends Plant Sci.

[CR12] Di Toppi LS, Gabbrielli R (1999). Response to cadmium in higher plants. Environ Exp Bot.

[CR13] EnsemblPlants. (n.d.). http://plants.ensembl.org. Accessed 23 Sept 2019.

[CR14] Fitz WJ, Wenzel WW (2002). Arsenic transformations in the soil-rhizosphere-plant system: fundamentals and potential application to phytoremediation. J Biotechnol.

[CR15] Guan C, Ji J, Li X, Jin C, Wang G (2016). LcMKK, a MAPK kinase from Lycium Chinense, confers cadmium tolerance in transgenic tobacco by transcriptional upregulation of ethylene responsive transcription factor gene. J Genet.

[CR16] Hall JL, Williams LE (2003). Transition metal transporters in plants. J Exp Bot.

[CR17] Hamel LP, Nicole MC, Sritubtim S (2006). Ancient signals: comparative genomics of plant MAPK and MAPKK gene families. Trends Plant Sci.

[CR18] Hayashi S, Kuramata M, Abe T, Takagi H, Ozawa K, Ishikawa S (2017). Phytochelatin synthase OsPCS1 plays a crucial role in reducing arsenic levels in rice grains. Plant J.

[CR19] Heo EB, Yoo JM, Lee WD, Chu SH, Kim KW, Cho YH, Park YJ (2017). Integrated genome-wide association studies to dissect natural variation for magnesium ion contents in rice germplasm. Korean J Breed Sci.

[CR20] Herawati N, Suzuki S, Hayashi K, Rivai IF, Koyama H (2000). Cadmium, copper, and zinc levels in rice and soil of Japan, Indonesia, and China by soil type. Bull Environ Contam Toxicol.

[CR21] Ishimaru Yasuhiro, Suzuki Motofumi, Tsukamoto Takashi, Suzuki Kazumasa, Nakazono Mikio, Kobayashi Takanori, Wada Yasuaki, Watanabe Satoshi, Matsuhashi Shinpei, Takahashi Michiko, Nakanishi Hiromi, Mori Satoshi, Nishizawa Naoko K. (2006). Rice plants take up iron as an Fe3+-phytosiderophore and as Fe2+. The Plant Journal.

[CR22] Ishimaru Y, Takahashi R, Bashir K, Shimo H, Senoura T, Sugimoto K, Ono K, Yano M, Ishikawa S, Arao T, Nakanishi H, Nishizawa NK (2012). Characterizing the role of rice NRAMP5 in manganese, iron and cadmium transport. Sci Rep.

[CR23] Jonak C, Nakagami H, Hirt H (2004). Heavy metal stress. Activation of distinct mitogen-activated protein kinase pathways by copper and cadmium. Plant Physiol.

[CR24] Jonak C, Okresz L, Bogre L, Hirt H (2002). Complexity, cross talk and integration of plant MAP kinase signalling. Curr Opin Plant Biol.

[CR25] Kang DW, Kim DY, Yoo JH, Park SW, Oh KS, Kwon OK, Baek SH, Kim WI (2018). Effect of soil amendments on arsenic reduction of Brown Rice in Paddy fields. Korean J Soil Sci Fert..

[CR26] Kang HM, Zaitlen NA, Wade CM, Kirby A, Heckerman D, Daly MJ, Eskin E (2008). Efficient control of population structure in model organism association mapping. GENETICS.

[CR27] Kim MS, Kim WI, Lee JS, Lee GJ, Jo GL, Ahn MS, Choi SC, Kim HJ, Kim YS, Choi MT, Moon YH, Ahn BK, Kim HW, Seo YJ, Lee YH, Hwang JJ, Kim YH, Ha SK (2010). Long-term monitoring study of soil chemical contents and quality in Paddy fields. Korean J Soil Sci Fert.

[CR28] Kögel-Knabner I, Amelung W, Cao Z, Fiedler S, Frenzel P, Jahn R, Kalbitz K, Kölbl A, Schloter M (2010). Biogeochemistry of paddy soils. Geoderma.

[CR29] Lee S, An G (2009). Over-expression of OsIRT1 leads to increased iron and zinc accumulations in rice. Plant Cell Environ.

[CR30] Lee SB, Kim KW, Yoon MY, Kim GJ, Yoo JH, Kim WI, Moon BC, Park SW, Park YJ (2016). Genome-wide association study for cadmium contents of temperate japonica varieties. Korean J Breed Sci.

[CR31] Leustek T, Saito K (1999). Sulfate transport and assimilation in plants. Plant Physiol.

[CR32] Limon-Pacheco JH, Hernandez NA, Fanjul-Moles ML, Gonsebatt ME (2007). Glutathione depletion activates mitogen-activated protein kinase (MAPK) pathways that display organ-specific responses and brain protection in mice. Free Radic Biol Med.

[CR33] Lin CY, Trinh NN, Fu SF, Hsiung YC, Chia LC, Lin CW, Huang HJ (2013). Comparison of early transcriptome responses to copper and cadmium in rice roots. Plant Mol Biol.

[CR34] Liu J, Li K, Xu J, Liang J, Lu X, Yang J, Zhu Q (2003). Interaction of cd and five mineral nutrients for uptake and accumulation in different rice cultivars and genotypes. Field Crops Res.

[CR35] Liu J, Qian M, Cai G, Yang J, Zhu Q (2007). Uptake and translocation of cd in different rice cultivars and the relation with cd accumulation in rice grain. J Hazard Mater.

[CR36] Liu JG, Liang JS, Li KQ, Zhang ZJ, Yu BY, Lu XL, Yang JC, Zhu QS (2003). Correlations between cadmium and mineral nutrients in absorption and accumulation in various genotypes of rice under cadmium stress. Chemosphere.

[CR37] Ma JF, Yamaji N, Mitani N, Xu XY, Su YH, McGrath SP, Zhao FJ (2008). Transporters of arsenite in rice and their role in arsenic accumulation in rice grain. PNAS.

[CR38] Marin AR, Masscheleyn PH, Patrick WH (1993). Soil redox-pH stability of arsenic species and its influence on arsenic uptake by rice. Plant Soil.

[CR39] McGrath SP, Zhao FJ, Lombi E (2001). Plant and rhizosphere processes involved in phytoremediation of metal-contaminated soils. Plant Soil.

[CR40] Mendoza-Cozatl D, Loza-Tavera H, Hernandez-Navarro A, Moreno-Sanchez R (2005). Sulfur assimilation and glutathione metabolism under cadmium stress in yeast, protists and plants. FEMS Microbiol Rev.

[CR41] Mitani-Ueno N, Yamaji N, Zhao FJ, Ma JF (2011). The aromatic/arginine selectivity filter of NIP aquaporins plays a critical role in substrate selectivity for silicon, boron, and arsenic. J Exp Bot.

[CR42] MOE (Minister of Environment, Korea) (2010) Soil environment conservation Act. Ministry of Environment, Republic of Korea

[CR43] Morishita T, Fumoto N, Yoshizawa T, Kagawa K (1987). Varietal differences in cadmium levels of rice grains of japonica, Indica, Javanica, and hybrid varieties produced in the same plot of a field. Soil Sci Plant Nutr.

[CR44] NAAS (National Academy of Agriculture Science) (2010) Analysis methods for soil chemical properties. National Academy of Agriculture Science, Republic of Korea Publication No. 11–1390802–000282-01

[CR45] Nakanishi H, Ogawa I, Ishimaru Y, Mori S, Nishizawa NK (2006). Iron deficiency enhances cadmium uptake and translocation mediated by the Fe2+ transporters OsIRT1 and OsIRT2 in rice. Soil Sci Plant Nutr.

[CR46] Nevo Y, Nelson N (2006). The NRAMP family of metal-ion transporters. Biochim Biophys Acta.

[CR47] Ogawa I, Nakanishi H, Mori S, Nishizawa NK (2009). Time course analysis of gene regulation under cadmium stress in rice. Plant Soil.

[CR48] Pinson SRM, Tarpley L, Yan W, Yeater K, Lahner B, Yakubova E, Huang XY, Zhang M, Guerinot ML, Salt DE (2015). Worldwide genetic diversity for mineral element concentrations in rice grain. Crop Sci.

[CR49] Rahman A, Nahar K, Hasanuzzaman M, Fujita M (2016). Manganese-induced cadmium stress tolerance in rice seedlings: coordinated action of antioxidant defense, glyoxalase system and nutrient homeostasis. C R Biol.

[CR50] Rao KP, Richa T, Kumar K, Raghuram B, Sinha AK (2010). In silico analysis reveals 75 members of mitogen-activated protein kinase kinase kinase gene family in rice. DNA Res.

[CR51] Reyna NS, Yang Y (2006). Molecular analysis of the rice MAP kinase gene family in relation to *Magnaporthe grisea* infection. Mol Plant-Microbe Interact.

[CR52] Sakai H, Lee SS, Tanaka T (2013). Rice annotation project database (RAP-DB): an integrative and interactive database for rice genomics. Plant Cell Physiol.

[CR53] Sasaki A, Yamaji N, Yokosho K, Ma JF (2012). Nramp5 is a major transporter responsible for manganese and cadmium uptake in rice. Plant Cell.

[CR54] Shimo H, Ishimaru Y, An G, Yamakawa T, Nakanishi H, Nishizawa NK (2011). Low cadmium (LCD), a novel gene related to cadmium tolerance and accumulation in rice. J Exp Bot.

[CR55] Stohs SJ, Bagchi D (1995). Oxidative mechanisms in the toxicity of metal ions. Free Radic Biol Med.

[CR56] Stranger BE, Montgomery SB, Dimas AS, Parts L, Stegle O, Ingle CE, Sekowska M, Smith GD, Evans D, Gutierrez-Arcelus M, Price A, Raj T, Nisbett J, Nica AC, Beazley C, Durbin R, Deloukas P, Dermitzakis ET (2012). Patterns of Cis regulatory variation in diverse human populations. PLoS Genet.

[CR57] Suzuki N, Koizumi N, Sano H (2001). Screening of cadmium-responsive genes in *Arabidopsis thaliana*. Plant Cell Environ.

[CR58] TAKAHASHI RYUICHI, ISHIMARU YASUHIRO, SHIMO HUGO, OGO YUKO, SENOURA TAKESHI, NISHIZAWA NAOKO K., NAKANISHI HIROMI (2012). The OsHMA2 transporter is involved in root-to-shoot translocation of Zn and Cd in rice. Plant, Cell & Environment.

[CR59] Ueno D, Koyama E, Yamaji N, Ma JF (2011). Physiological, genetic, and molecular characterization of a high-cd-accumulating rice cultivar, Jarjan. J Exp Bot.

[CR60] Uraguchi S, Kamiya T, Sakamoto T, Kasai K, Sato Y, Nagamura Y, Yoshida A, Kyozuka J, Ishikawa S, Fujiwara T (2011). Low-affinity cation transporter (OsLCT1) regulates cadmium transporter into rice grains. PNAS.

[CR61] Vaid N, Pandey PK, Tuteja N (2012). Genome-wide analysis of lectin receptor-like kinase family from Arabidopsis and rice. Plant Mol Biol.

[CR62] Wang J, Yu H, Xie W, Xing Y, Yu S, Xu C, Li X, Xiao J, Zhang Q (2010). A global analysis of QTLs for expression variations in rice shoots at the early seedling stage. Plant J.

[CR63] Xia J, Yamaji N, Ma JF (2013). A plasma membrane-localized small peptide is involved in rice aluminum tolerance. Plant J.

[CR64] Xia L, Zou D, Sang J, Xu X, Yin H, Li M, Wu S, Hu S, Hao L, Zhang Z (2017). Rice expression database (RED): an integrated RNA-Seq-derived gene expression database for rice. J Genet Genomics.

[CR65] Xu W, Dai W, Yan H, Li S, Shen H, Chen Y, Xu H, Sun Y, He Z, Ma M (2015). Arabidopsis NIP3;1 plays an important role in arsenic uptake and root-to-shoot translocation under Arsenite stress conditions. Mol Plant.

[CR66] Yamaji N, Xia J, Mitani-Ueno N, Yokosho K, Ma JF (2013). Preferential delivery of zinc to developing tissues in Rice is mediated by P-type heavy metal ATPase OsHMA2. Plant Physiol.

[CR67] Yamazaki Shinichi, Ueda Yosuke, Mukai Aya, Ochiai Kumiko, Matoh Toru (2018). Rice phytochelatin synthases OsPCS1 and OsPCS2 make different contributions to cadmium and arsenic tolerance. Plant Direct.

[CR68] Yang TP, Beazley C, Montgomery SB, Dimas AS, Gutierrez-Arcelus M, Stranger BE, Deloukas P, Dermitzakis ET (2010). Genevar: a database and Java application for the analysis and visualization of SNP-gene associations in eQTL studies. Bioinformatics.

[CR69] Yeh CM, Chien PS, Huang HJ (2007). Distinct signalling pathways for induction of MAP kinase activities by cadmium and copper in rice roots. J Exp Bot.

[CR70] Yu J, Pressoir G, Briggs WH, Bi IV, Yamasaki M, Doebley JF, McMullen MD, Gaut BS, Nielsen DM, Holland JB, Kresovich S, Buckler ES (2006). A unified mixed-model method for association mapping that accounts for multiple levels of relatedness. Nature Geneticsvolume.

[CR71] Yuan L, Yang S, Liu B, Zhang M, Wu K (2012). Molecular characterization of a rice metal tolerance protein, OsMTP1. Plant Cell Rep.

[CR72] Zhang Z, Ersoz E, Lai C-Q, Todhunter RJ, Tiwari HK, Gore MA, Bradbury PJ, Yu J, Arnett DK, Ordovas JM, Buckler ES (2010). Mixed linear model approach adapted for genome-wide association studies. Nat Genet.

