# Supplementary figures and images for: Functional Haplotype and eQTL Analyses of Genes Affecting Cadmium Content in Cultivated Rice
Source: Rice (N Y). 2019 Nov 21;12:84. doi: 10.1186/s12284-019-0340-8 (PMC6872708; doi:10.1186/s12284-019-0340-8)

**a**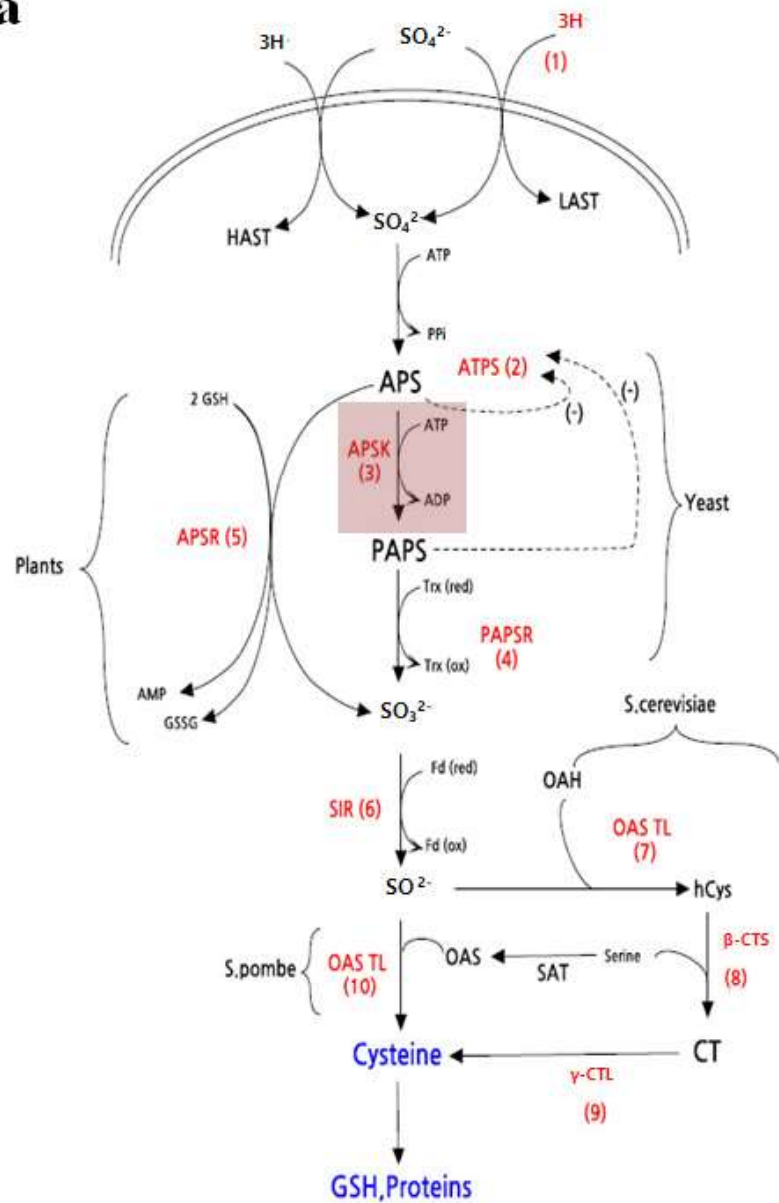**b**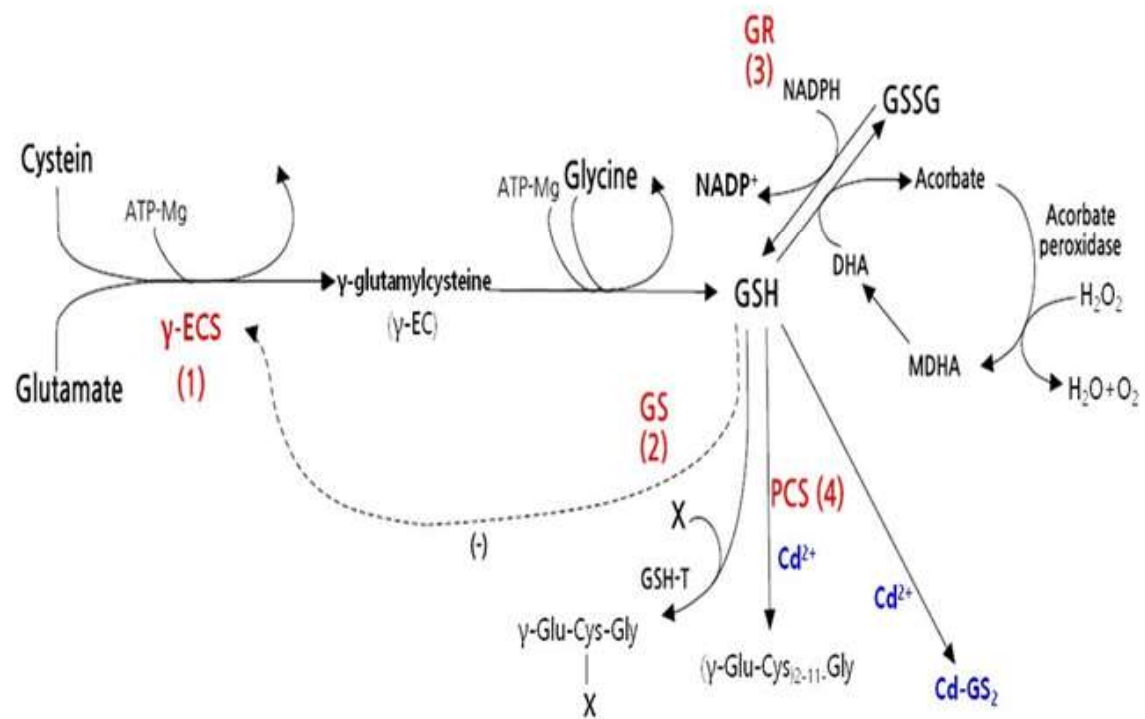

Supplement: Supplementary file 1 — Additional file 1: Figure S1. Glutathione (GSH) biosynthetic process for Cd uptake in plants. Amplitude-phase shift keying (APSK) and phosphoadenosine phosphosulfate reductase (PAPSR) involved in GSH synthesis. a Sulfate assimilation pathway and biosynthetic process of cysteine; b GSH synthesis and ROS processing. *This figure is a reconstruction of the original source (Mendoza-Cozatl et al. 2005). [file 12284_2019_340_MOESM1_ESM.pdf]
